# Faculty development and institutional readiness for incorporating AI in health professions education- A narrative review

**DOI:** 10.3389/fdmed.2026.1874590

**Published:** 2026-07-22

**Authors:** Prathibha Prasad, Mahinour Amin, Elizabeth Fitriana Sari, Lovely M. Annamma, Jayaraj Narayanan, Dinesh Yasothkumar, Mehzabin Ahmed

**Affiliations:** 1 Department of Basic Medical and Dental Sciences, College of Dentistry, Ajman University, Ajman, United Arab Emirates; 2 Director of Teaching and Learning Center, Ajman University, Ajman, United Arab Emirates; 3 Adjunct Faculty, Dental Research Cell, Dr D. Y. Patil Dental College & Hospital, Dr D. Y. Patil Vidyapeeth (Deemed to be University), Pune, Maharashtra, India; 4 Consultant Oral and Maxillofacial Pathologist, Dentscope Advanced Dental Studio, Kilpauk, Chennai, India; 5 Biomedical Sciences Department, College of Medicine, Gulf Medical University, Ajman, United Arab Emirates

**Keywords:** artificial intelligence, dental education, ethics, faculty development, global perspectives, health professions education, higher education institutions

## Abstract

Artificial Intelligence (AI) is presently reshaping higher education in a rapid way, due to the high engagement of students with AI. The study's aim was to explore strategies driving transformation in higher education at large and particularly dental education. This article is a narrative review adopting a structured approach for literature search, study selection, and data extraction. A comprehensive search of electronic databases, including PubMed, Scopus, Google Scholar, and ScienceDirect for studies published between 2015 and 2025 was conducted. The review is discussed under the themes of transformative education, institutional readiness, including resistance to change, ethical considerations and global perspectives in health professions, and dental education. Further, transformative education explores faculty development, including micro-credentials and lifelong learning, a safe learning environment, infrastructure, and assessment. AI is well acknowledged in both health profession education and clinical practice. While GenAI has the potential to improve creativity, feedback, and collaborative learning, the issues of plagiarism, assessment validity, and authorship transparency are also discussed. AI encounters limitations in gauging psychomotor dexterity and clinical reasoning. Ethical concerns about algorithmic prejudice, data privacy, monitoring, and the deterioration of human-centered learning are also included. To conclude, transformative strategies for institutional readiness to be considered under the domains of governance and policy, curriculum integration and faculty training, academic integrity and assessment reforms, and leadership and change management to ensure proper integration of AI and to prepare competent, adaptable, and socially responsible health professionals. Maintenance of human oversight at the center of higher education is essential, and universities should treat AI as a catalyst for modernization rather than a threat.

## 1 Introduction

Artificial Intelligence (AI) is broadly defined as the simulation of human intelligence by systems capable of perception, reasoning, learning, planning, and prediction (1). Generative AI (GenAI) and Large Language Models (LLMs) are presently reshaping higher education in a way that is considered transformative as the internet's inception (2, 3). In health professions education (HPE), GenAI is used for several educational processes such as resource development and delivery, personalizing learning, assessments, providing feedback and formulating evaluation and to a lesser extend for curriculum design (4). Gen AI is perceived to be user friendly and is seen to be transformative by both faculty and students. Studies report that a large majority of university students (80%–86%) use AI tools for learning, such as creating resources, to seek feedback for communication skills and exercises in humanities, support their self-directed learning for exam preparation, as well as for research and publications for writing assistance, and data analysis (5–10). Educational chatbots using GenAI like ChatGPT are used by medical and dental students as virtual patients creating clinical scenarios allowing practice of history taking, diagnostic reasoning and treatment planning (11). These experiences were perceived to be more satisfying, less stressful and improved their motivation and engagement (12). A study comparing learning outcomes amongst dental students found better results among those who used ChatGPT for test preparation (13). Studies have shown that patients use GenAI, mostly ChatGPT and Google Gemini, to obtain health information, seek explanation of symptoms experienced and their self-management. The most common reasons cited are easy accessibility, speed of response and a perception of empathy in the results received (14). However, the extent of its use is determined by the educational level, field of expertise and awareness of AI tools and its use (15).

However, some unsettling limitations and challenges faced with the use of GenAI are the generation of fake information, overdependence and academic dishonesty. The term “hallucination” is used to describe the phenomenon of misinformation with a clear explanation and fabrication of citations and references. This results in the propagation of the deceptive or biased information, especially if the student does not verify the authenticity of the generated content (16, 17). Additionally, the information generated in response to prompts in some areas may be biased or based on old information or errors in training, resulting in misinformation; the latest research may add a layer of clarification or even contradict an existing concept (7, 18, 19). Overreliance on GenAI without actually investing efforts to learn or complete assigned tasks reduces the effectiveness of the learning exercise (19). Academic integrity, an important criterion for professionalism, may be breached when students use AI to cheat in exams or plagiarize academic work (20, 21). Some researchers also argue that the use of GenAI removes the human perspective from the learning experience due to its capabilities being limited to a textual format and its inability to identify or interpret nonverbal cues, which in some students may have a detrimental effect (16, 22). A study on measurable impacts of AI based strategies on educational outcomes in HPE, showed that the strength of evidence is poor, and there is a lack of guidelines and criteria to evaluate the impact (23). Further, it suggests that AI integration should be attempted in blended learning environments to add the benefit of human interaction.

With increasing use of GenAI in education, another growing concern regarding its negative effect on the learning process is the limited use of critical reasoning and problem-solving skills, because of AI dependency and lack of human interaction, crucial for communication and patient interactions and a student's transition to a professional (24–26). AI is progressively becoming integrated into current dental workflows, particularly in diagnosis, treatment design, and predictive models of disease (27). These evolving technologies are transforming clinical practice, alongside a shift in the core competencies demanded by future dental practitioners (28).

Consistent with the changing milieu of dental education and practice, integration of curricular reforms and innovations, in teaching learning methods, assessments and evaluations, into existing systems is essential to train future-ready educators and students for a progressively digitalized and data-intensive healthcare setting (29). These efforts should be guided by revised policies for student training and academic progression (30, 31). Faculty and institutional preparedness are vital for this shift. Faculty should possess core AI understanding and weave the strategies of the use of AI tools into the curriculum, its delivery and evaluation. Institutionally, investment in infrastructure, regulatory frameworks, and collaborative initiatives across disciplines is necessary for responsible and effective implementation (30). The adoption of AI within dental academia extends beyond content addition; it mandates a broader shift from knowledge-centric teaching to competency-focused paradigms that incorporate digital tools. Students must acquire not just technical expertise but also data literacy, skills for critical assessment, and an insight into AI's ethical challenges (32). Global evaluations point to a significant variation in institutional capabilities, funding and resources, and pedagogical focus (33). AI integration in dental education is inconsistent globally. Lack of tailored locale-specific strategies may widen disparities in pedagogical resources and expertise, compromising educational equity and clinical care efficacy (34). The aim of the study was to explore strategies driving transformation in higher education at large and dental education, particularly. It devotes special attention to faculty preparedness and barriers to adoption, the imperative for revising assessment practices to protect academic integrity and strategies to sustain stakeholder trust in education frameworks.

This narrative review aims to synthesize current evidence on integrating AI in health professions education with an emphasis on dental education. It discusses implementation strategies for transformative education focusing on institutional readiness, faculty development including micro-credentials and lifelong learning, safe learning environment, infrastructure, assessment, ethical considerations, and global perspectives, while identifying key gaps to inform future research and practice.

## 2 Methodology

**Figure 1:**
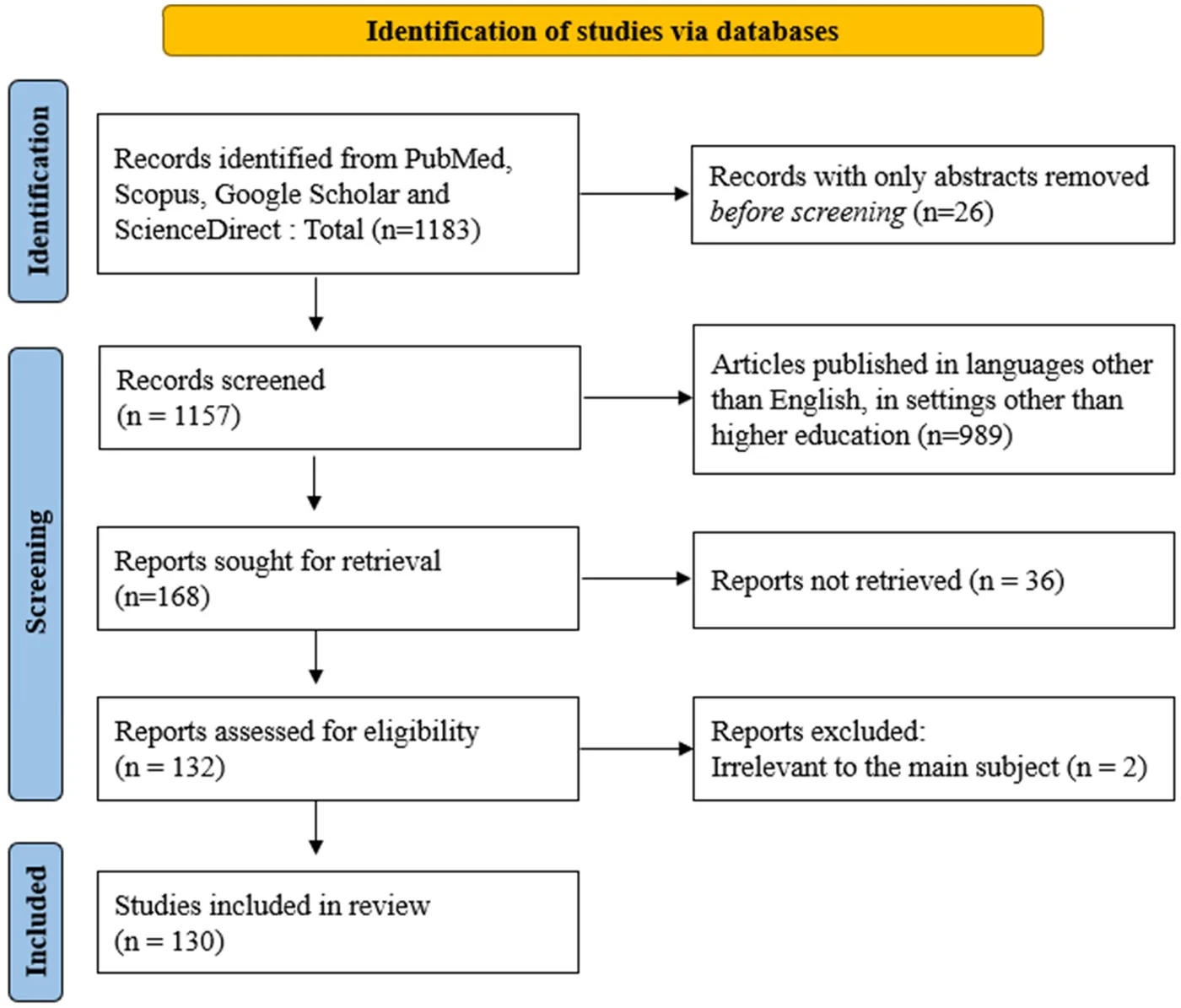
Flow chart of study selection.

This article is a narrative review adopting a structured approach for literature search, study selection, and data extraction, to enhance transparency and methodological clarity. (Figure 1) Given the heterogeneity of the included studies, the findings were synthesized narratively (35, 36).

### 2.1 Search strategy

The authors conducted a comprehensive search of electronic databases, including PubMed, Scopus, Google Scholar, and ScienceDirect, for studies published between 2015 and 2025. To complement the electronic search, reference lists of included studies were also screened to identify additional eligible publications that might not have been retrieved through the database search.

The search strategy was developed using appropriate Medical Subject Headings (MeSH) and free-text terms, such as “Artificial intelligence,” “Digital technologies,” “Transformative medical education,” “Institutional readiness,” “Faculty development”, “AI applications,” “Dental education” and “Dental practice” Boolean operators and database-specific filters were applied where applicable to refine and maximize the sensitivity of the search.

Inclusion and Exclusion criteria: The eligibility criteria for inclusion was based on the year of publication, to comprise studies from 2015 to 2025. Articles published in English language and in settings of higher education were included. Studies of a diverse range of designs, including international policy guidelines published on official organizational websites, educational guides, empirical and interventional studies, case studies, surveys, review articles, and meta-analyses were considered. Publications on dental and health professions educational settings that evaluated AI-based tools, systems, or strategies which reported the educational outcomes and implementation processes.

Exclusion criteria were also clearly defined. Studies lacking a reported study of design or those using designs deemed inappropriate for the review were excluded. Similarly, non-peer-reviewed materials such as book chapters, editorials, and conference abstracts were excluded to ensure the reliability and scientific rigor of the evidence base. This structured approach ensured that only high-quality and methodologically sound studies were included, thereby strengthening the validity of the findings.

### 2.2 Study selection and data extraction

Following the literature search, retrieved articles were imported into Mendeley software, where duplicate records were identified and removed. As part of this narrative review, a structured screening approach was adopted to enhance transparency and consistency. The research team reviewed titles and abstracts to identify potentially relevant studies, which were then subjected to full-text assessment for eligibility. Decisions regarding inclusion were made through discussion and consensus among the authors. While efforts were made to ensure a structured and transparent selection process, the approach was applied within the context of a narrative review rather than a formal systematic review.

### 2.3 Data extraction

Data extraction from all included studies was conducted meticulously using a structured data-charting approach. Extracted information included publication details (author, year, country, and study design), faculty development initiatives related to AI, faculty perceptions and attitudes toward AI adoption, institutional readiness factors, technological infrastructure, governance and policy considerations, ethical and regulatory issues, curricular integration strategies, implementation challenges, and recommendations for AI integration within health professions education (37). This manuscript was conducted as a narrative review, with multiple authors contributing to different thematic sections based on their expertise. The collaborative approach facilitated comprehensive thematic synthesis and interdisciplinary interpretation of the evidence. All disagreements were resolved through discussion among the authors. The screening process followed four sequential stages: identification, duplicate removal, title and abstract screening, and full-text eligibility assessment. Articles meeting all eligibility criteria were subsequently included in the final narrative synthesis. Although a risk-of-bias tool was not applied, methodological quality and relevance were evaluated (38, 39).

## 3 Transformative education

Transformative education is defined as cognitive and psychological redirection, forging development of opinions and alteration in practices based on newly gained perspectives (40). It challenges prior knowledge, values, customs and expectations and settling such dilemmas by discussions and research, along with emotional and ethical engagement (41). AI has emerged as a major transformative force and the structural shift in medical education witnessed today is principally driven by redefinition of competency outcomes necessitating the integration of AI in the curriculum design and implementation (42, 43). AI has influenced use of innovative digital applications and tools and changing healthcare needs (43). Using AI tools to personalize learning especially clinical reasoning and communication skills improves the learner engagement. Learner engagement is defined by the extent of active participation of a learner, cognitively, behaviorally and emotionally, in educational activities (44). Such continuous enhancement of skills, competencies and professional identity coupled with motivation to engage in life long learning is referred to as professional development. In the context of AI, it refers to acquiring AI literacy, upholding ethical standards, collaborating and adapting to the newer advancements. AI integrated medical training empowers learners with skills and abilities to employ technologies to deliver patient-centered care that is individualized, compassionate, evidence driven and ethical (45). It has been demonstrated that using AI improves learning outcomes, motivation, and students' attitudes toward learning, all of which have a favorable effect on student performance.

Educators can now become facilitators of learning, be decision-makers, and lead innovations, which necessitates a more reflective mindset, embracing principles of a safe learning environment and capable of adopting transformation through an evidence-informed, strategic, and collaborative approach (46, 47). However, such transformation must be driven by faculty development (FD); it not only builds consensus and generates support for faculty but also motivates them to implement and sustain the change by institutionalizing it as a culture (48, 49). Thus, to make transformative education sustainable, three developmental targets must be addressed: faculty development, including micro-credentials and lifelong learning, safe learning environment, and infrastructure.

### 3.1 Faculty development

Faculty development programs (FDPs) are defined as programmed activities that provide an institution and its faculty with the knowledge of complex determinants of teaching and learning and hone their skills to fulfil their roles (50–52). Institutions with effective communities of practice promote strong educator identity formation and adoption of good practices during FDPs (53). FD may be institutional initiatives or be undertaken by faculty, sometimes as a sabbatical leave. Fellowship programs, MCs, short certification courses, workshops or CME seminars by experienced peers, guidance programs by specialists or as an assessment of faculty for quality assurance. FD by means of online resources or face-to-face programs is a critical mediator for an efficient learning environment (LE) (54). FDPs are driven by institutional culture, technological change, and individual motivation, and therefore, need to be continuous and adaptive to the emerging needs, for sustainable, transformative change, without which there is a risk of widening the gap between technological advancements and faculty preparedness (53, 55). The areas to be targeted in FD programs (FDPs) should encompass professional, instructional, leadership and organizational development and be tailored comprehensively to address specific needs and strengthen the multiple roles of the educators (52, 56).

In technology-enhanced educational systems today, especially with the increasing use of AI, the most pressing issues facing educators is the development of competencies such as data literacy, designing and implementing technological tools and applications in teaching and learning, while observing the ethical considerations (57). For AI integration in HPE, FDPs should target pedagogy, assessments, feedback and role modelling. Pedagogy in AI in HPE is defined by the theoretical basis of the curricular changes, strategies for personalized tutoring, and adaptive learning (58), Progressive exploration and application. to clinical decision-making, running diagnostics, administration of healthcare systems (HCS), and contributing to innovations in the learning environment, make AI literacy a core competency expected of health professionals (HPs)and educators (28, 59). It is important for institutions to address AI literacy through structured pathways following a longitudinal development trajectory when designing scalable and transformative FDPs to equip educators to be future-ready (55). Faculty training to design formative assessments employing AI that are customized to learner needs, measuring their progress and offering regular and timely feedback to learners. Further the use of AI enabled simulations and video analytics can be use to assess clinical skills and reasoning. Machine learning models have also been used to evaluate assignments (42). An interesting application of AI-powered avatars to train educators in giving feedback renders an engaging and realistic approach to FDPs (60). Role modelling in FD for AI integration in HPE refers to advocating the use of AI to fellow faculty increases the likelihood of adoption of its use (61) AI generated videos used for faculty training are found to be engaging in spite of their unrealistic appearance (62).

The Digital Education Council's AI Literacy Framework for higher education institutes suggests five dimensions with defined competency levels for each. The dimensions increase in their complexity and depth of learning, starting with a basic understanding of AI and data processing, progressing to critical thinking and decision making about the use and application of AI, ethical and responsible use of AI by HPs, human centered practices, using emotional intelligence and creativity, and finally addressing the development of expertise and leadership in AI application in HCS and education (34). In health professions education, these proficiencies may be applied to curriculum design, pedagogical strategies, assessments, student support, faculty development, research, innovative and transformational operations, while considering the biases, ethical governance, and maintenance of a humanistic approach (63). FD workshops, continuous medical education (CME) activities, and micro-credentials (MCs) serve two essential functions for continuous professional development (CPD). Firstly, they foster lifelong learning, adaptability, reflective practice, and professional identity formation among educators; and secondly, they facilitate institutional alignment to educational goals, accreditation standards, and healthcare priorities (64, 65). They instill confidence to adapt and integrate evidence-based pedagogical approaches and respond to evolving expectations of the learners (55). Importantly, planning for FDPs must also address barriers such as time constraints, lack of institutional support, and limited access to resources, particularly in low-resource settings (65). Thus, FD is a cornerstone of transformative medical education, especially in newer educator competencies, such as digital tools and AI, preparing them to lead in an era of rapid technological and educational change.

#### 3.1.1 AI and micro-credentials

Micro-credentials, alternatively called digital badges, micro-certifications, are short courses or training modules that enable the learner to master a specific topic or skill, affording personalized professional development (66–69).

AI is revolutionizing higher education, especially dental education. Using AI in micro-credentialing affords flexible skill development through adaptable content and tailored learning pathways. This allows educational institutions to customize faculty training to achieve competency in use of AI in health professions and dental education (70). Vald et al. proposed an AI literacy framework for healthcare micro-credentials that maps competency requirements across roles and facilitates collaborative curriculum building for training. Additional strengths of the framework include its wide applicability and collaborative curriculum design. Increasing adoption of micro-credentials and digital badges, supports scalable AI literacy development in healthcare (71, 72)..

Study recommends that dental schools should build a vertical AI competency framework across preclinical and clinical years (73, 74). In dental education, AI literacy can be fostered through scalable, role-specific micro-credentials that support curricular integration, faculty development, clinical training, and the responsible use of AI. This approach is well suited to continuing professional development for dentists, dental hygienists, and faculty members, and it complements dental AI curricula that emphasizes governance, explainability, data privacy, and accountability (75)..

While traditional CPDs are delivered in an episodic manner, they are found to have a limited benefit on clinical performance and patient outcomes. Traditional CPDs are time bound activities that address large groups of HPs, offering no individualized feedback or skill mastery. MCs provide skill specific, content relevant activities that are flexible, assessed, and certifiable (76, 77). Multiple MCs on a broader topic may be added in an incremental manner, without placing a burden on the constrained time of practicing HPs, to address emerging areas of professional practice, such as digital health and teaching skills (78). The goal is to build a system that can strengthen the role of context of AI in teaching and learning (79).

### 3.2 Safe learning environments

LE includes physical, social, and psychological elements that determine the learning, professional identity formation, and the overall culture of the institution (80). Safe learning environments (SLEs) are LEs that physically, socially, and psychologically exert a positive effect on student experience in formal, informal, and hidden curricula (81). As learning is situated, relational and contextual, LE must also be dynamic and modifiable and not just a passive fixed constituent of the curriculum (82, 83). Psychological safety is defined as a belief that the environment is safe and supportive to make mistakes while learning (84). Studies using AI driven avatars embedded in simulation models to provide personalized feedback in real time adapting as the student progresses through the simulation. Preliminary studies using such avatars has shown to improve procedural skills and diagnostic accuracy with a potential to employ the models for training and assessing multidisciplinary teams as well as for patient education (85, 86). The results of a study on training dental professionals using AI-enabled simulation promotes safe, ethical, and learner-centered clinical education. Chatbots enable practice interviewing pediatric patients in a risk-free, standardized setting without compromising patient safety or well-being (87).

The most visible outcomes of a conducive, nonthreatening, and respectful LE are academic and professional achievements of students, student behavior, and satisfaction (59, 88). Environments that embrace diversity, promote inclusivity and equality empower students to actively engage, collaborate and seek feedback to achieve the set objectives confidently without feeling overwhelmed, anxious and burnt out (89, 90) Inclusivity is defined as embracing students of diverse nature in terms of culture, socioeconomic status and experience creating a respectful and safe LE (91). The favorable attributes that define SLE, additionally, forge a strong relationship between and among the teachers and students, underlined by a healthy rapport, open channels of communication, mutual trust, ethical practices and professional identity formation within the community of practice (92). Such SLEs adapt to learners' needs and accommodate diverse talents and learning styles, while gradually raising the bar of expectations for the learner to achieve higher (92). SLE promote professional identity formation is a dynamic developmental process by which learners imbibe and internalize the values, demonstrate professional behaviors and maintain ethical standard of medical practice as they learn to “think, act and feel like physicians” (93). GenAI like Chat GPT can be used to mentoring HPE students to strengthen their PIF (94). CBME has defined the bar for learner outcomes; digital transformations and increasing integration of workplace-based learning adds to the complexity of the learning environment (LE). Digital and AI learning and assessment platforms, learning analytics and simulation laboratories have introduced a digital revolution in LEs (30, 95, 96). Undoubtedly, these digital and pedagogical innovations have increased the flexibility in learning and assessments and curriculum implementation. However, variability in resources may interfere with the alignment of curriculum and the LE. Another challenge faced with affording inclusivity in higher education is the acceptance by peers and self-perception of the “differently abled” student, putting students at risk of violence and psychological trauma, increased starkly with the inexperience of faculty in dealing with such situations (97). A study comparing patient safety competency frameworks that guide the curricular initiative to define the principles of quality patient care and safety. The frameworks compared include those by the WHO, Australian, United Kingdom, Canadian, and Nordic committees. While regional frameworks are contextual and reflect local priorities, principles of professionalism, patient safety, teamwork and embedding these in educational systems with an emphasis on psychological safety for learners to discuss and learn from mistakes, using simulations to develop clinical skills, promoting constructive feedback and reflective learning. The WHO model provides a universal framework and is suitable for adaptation across countries and across education for all health professions including dental education (98). Therefore, a propitious alignment between a conducive learning environment, infrastructure, and workforce development is essential for the delivery of quality medical education.

### 3.3 Infrastructure

Infrastructure refers to the technical and administrative organization of resources that will promote the integration and sustainability of AI in HPE. It includes learning spaces, equipment, software, network connectivity, data management tools and systems, cybersecurity systems, technical support, institutional guidelines and policies (99). Learning spaces may be physical, virtual or hybrid and support collaborative learning, simulation based training and digital learning environments conducive to immersive learning experiences. In the context of AI integration in HPE these spaces could be smart classrooms, simulation labs, virtual learning spaces and immersive digital environments.

#### 3.3.1 Digital applications in dental education

Digital platforms refer to online learning management systems, applications and educational technologies that can be integrated to facilitate the curriculum delivery, management and assessment and evaluation of HPE (100). Advanced digital technologies in dental education have been broadly explored in recent literature, demonstrating measurable improvements in learning efficiency, student performance, and assessment quality (101). The use of AI personalizes learning through learning analytics, virtual reality (VR), smart classrooms, conversational agents, and customized assessment and feedback systems (102). Studies have acknowledged the use of personalized digital learning technology as a useful method with several benefits. In addition to performing noticeably better on tests, students who used the adaptive system reported feeling more confident about their learning progress and experiencing less mental strain (103). Studies have shown that AI tools adapting to the learning styles of students significantly improved academic performance, with student scores rising by as much as 15% to 30% (104). However, the efficacy of the GenAI tool used for gathering information, consolidating knowledge, and communicating clinical reasoning during post-clinical discussions is found to be variable and warrants a cautious approach (105, 106). AI platforms assess the cognitive levels, psychological states, and motivation levels, and offer opportunities for growth (107). A study revealed that the language-based model systems have shown accuracy ranging from 78.8% to 80.98% in dental examination responses, with advanced versions achieving up to 79.57% accuracy (104).

#### 3.3.2 AI applications in dental simulation and clinics

Simulation refers to the use of technology to replicate clinical scenarios, patient encounters and healthcare systems to facilitate experiential learning, deliberate practice, clinical decision making and competency development in a safe environment. Simulation-based learning in dentistry offers safe, rich, and scalable clinical experiences. In the context of AI, simulations using LLM models facilitate early development of clinical reasoning, diagnostic abilities and therapeutic decision making (108). Simulation provides risk-free environments to practice complex procedures in endodontics and implantology specialties. It supports the development of clinical reasoning, radiographic interpretation, and diagnostic skills through AI-generated interactive virtual patient interviews, simulations, and personalized learning pathways (109, 110). A review elaborates on the integration of AI in RL-driven haptic and VR simulations, deep-learning-supported virtual patient generation, and multimodal simulation environments (111). Evidence of effectiveness includes ChatGPT-assisted simulation (e.g., simulated patient interviews) significantly improved clinical communication, interviewing, and judgment (109). The AI-powered simulation enhanced the diagnosis of periapical lesions, periodontal disease, and oral pathology with accuracy rates exceeding 90% in some domains (111). Research on radiographic interpretation reports periapical lesion detection sensitivity of more than 93% to 95.3% and area under the curve (AUC) values of up to 0.96 (112, 113). Studies show that the pooled sensitivity and specificity for caries detection are 0.86 and 0.91, respectively, with an AUC of 0.94 (114). Human interpretation by experienced dental radiologists demonstrates strong diagnostic accuracy, although slightly lower sensitivity and specificity compared to rapid and objective feedback by software-based analysis, indicating its role as a supportive adjunct in caries detection (115).

Learning analytics is a methodical collection, analysis, interpretation and use of the derived data to monitor student performance, tailor instructions and support mechanisms to bolster learning needs and improve educational outcomes (116). Applying AI algorithms to predict outcomes, allows for preemptive remedial measures to improve outcomes. In dental education, the role of AI technologies is to achieve learning outcomes, enable personalized instruction, and ensuring objective assessment. These findings demonstrate excellent diagnostic ability, especially in the early detection of lesions. This facilitates prompt intervention and lessens the need for involved restorative procedures, supporting a preventive approach to care. Studies on periodontal diagnosis show that diagnostic accuracy ranges from 0.82 to 0.85, with sensitivity exceeding 90% for identifying alveolar bone loss and AUC values above 0.88 (117). The ability to produce reliable measurements improves the long-term tracking of disease progression. Long-term periodontal outcomes are enhanced, and treatment planning is therefore more accurate.

While orthodontic predictive models show accuracy rates of 92%–96% in identifying treatment requirements, evidence in treatment planning suggests accuracy exceeding 90% in identifying clinically relevant parameters (118, 119) these results show decreased subjectivity and increased consistency in complicated clinical situations. Digital tools for clinical and diagnostic skills training of students use AI-driven image analysis. CAD/CAM systems have shown improved reproducibility, shorter fabrication times, and occlusal discrepancies as low as 25.7 µm in restorative applications (120). These results show increased accuracy and consistency, which enhance clinical performance and patient-centered care. However, the challenges in adopting simulation include high start-up costs (VR hardware, software licensing), inadequacy of simulations as a replacement for real patient exposure, regular updates to ensure clinical realism, and current best practices (121).

**Figure 2:**
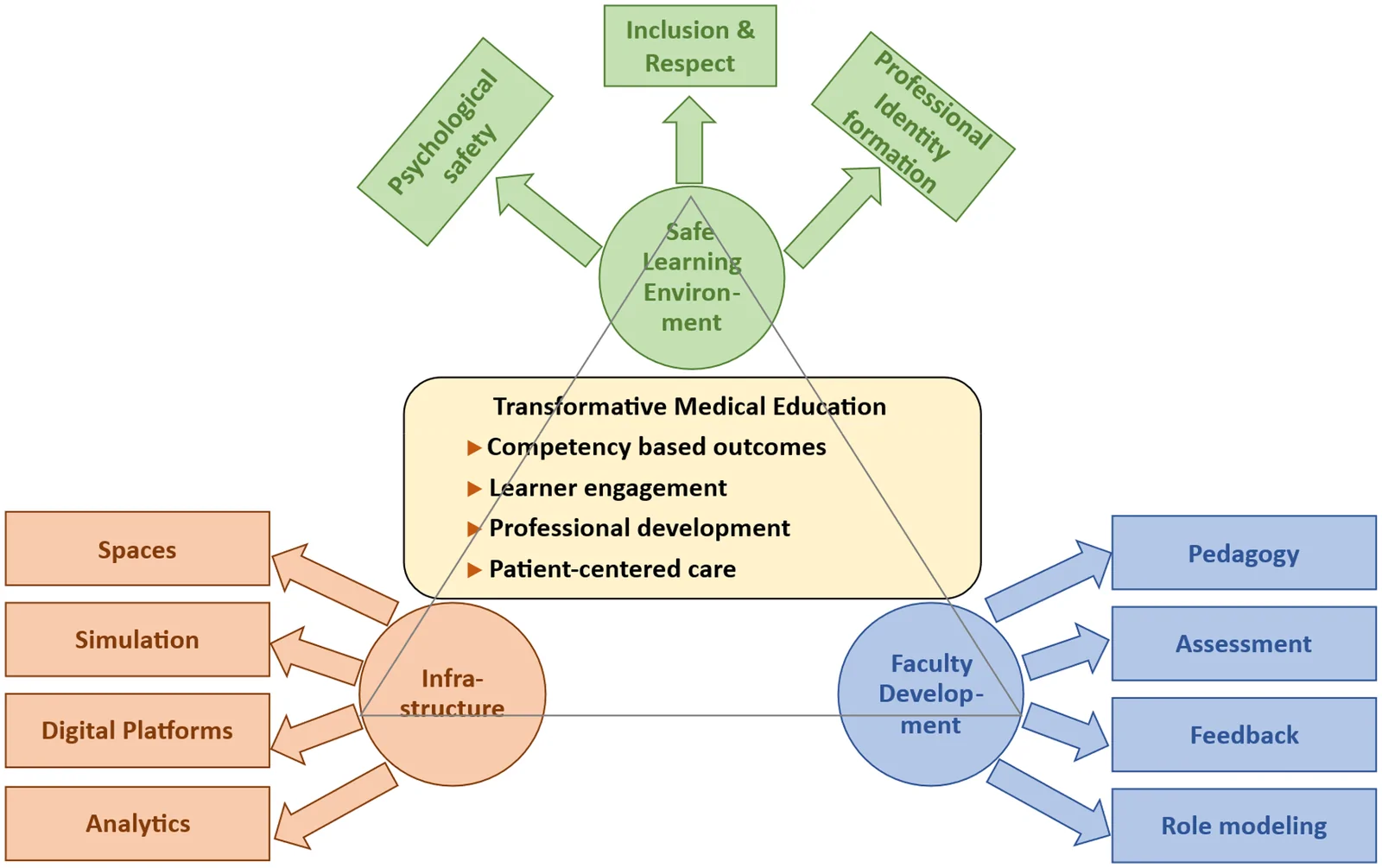
Determinants of sustainable transformative health professions education.

To conclude, it is critical to conceptualize the interdependence of the infrastructure development and deployment, faculty training, and SLE as components of institutional readiness for a coherent and sustainable educational system. (Figure 2).

## 4 Institutional readiness

**Figure 3:**
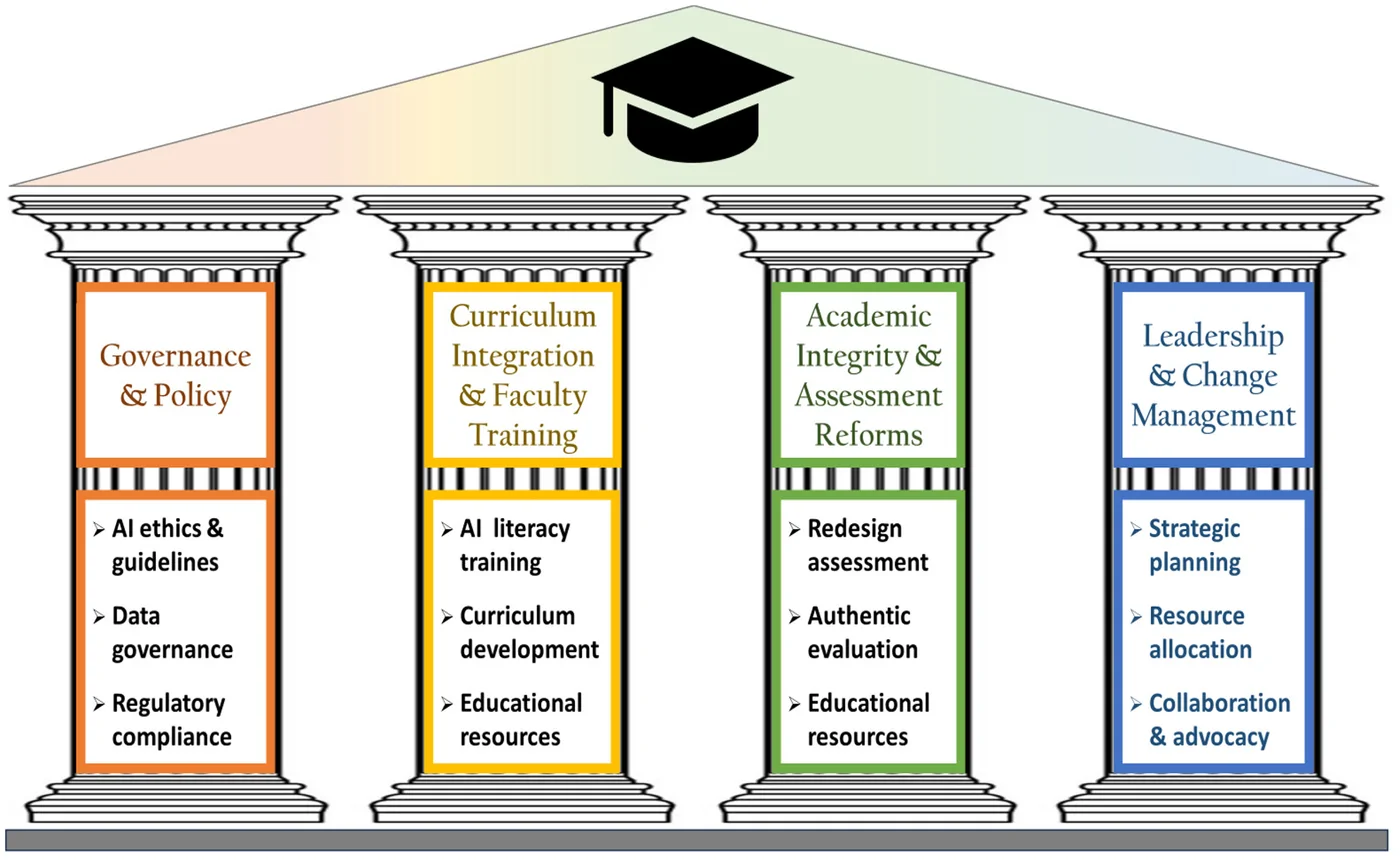
Domains of institutional readiness for AI integration in medical education.

Institutional readiness is the capacity of an institution to adopt and implement an educational change and is based on frameworks from CBME (70). In recent times, the increasing application of AI in the healthcare industry imposes additional competency requirements for the use of AI technology in clinical practice and research. This undoubtedly calls for integrating AI systems into medical training, optimizing institutional readiness to adopt and implement the change, and restoring the humanist approach to an otherwise automated patient care (122). Institutional readiness can be considered across four interrelated domains: governance and policy; faculty capacity and curriculum integration; academic integrity and assessment reforms; and leadership and strategic change management. (Figure 3) The role of the educational leadership is pivotal in making such a change sustainable without undermining academic standards and educational quality (6, 123, 124).

Governance and policy development domain sets into motion the institutional structures for AI adoption (125). Policies are defined as “*formal, authoritative statements that establish rules, responsibilities, and procedures*”. In contrast to guidelines which are recommended frameworks that may be employed in the implementation of the policy in a consistent manner. Policies are regulatory in nature and have legal consequences, while guidelines can be flexible to adapt to specific contexts. Institutional governing bodies approve policies while guidelines are formulated at a lower level such as department or committee (126). Governance models, at organizational level, should include the major stakeholders such as the leadership, faculty, administration and learners. Such bodies have regulatory, policy building and strategy planning functions (123). The strategies spelt out by governing bodies ensure support and infrastructure development, literacy and training, integration, fairness, transparency, trustworthiness, and accountability while highlighting the implications of improper AI adoption in healthcare systems (126–128). Such efforts ensure a uniform code of conduct for all healthcare institutions in a country (129–131). United Nations Educational, Scientific and Cultural Organization (UNESCO) recommendations provide a “universal framework of values, principles and actions to guide states in the formulation of their legislation, policies or other instruments regarding AI, consistent with international law.” These lay emphasis on formulating mechanisms to uphold transparency, accountability, and human oversight (132). Similarly, regulatory initiatives such as the European Union Artificial Intelligence Act and guidelines published as AMEE Guides outline details of appropriate provision and ethical use of AI in various sectors, including education (133, 134).

The second domain involves faculty development (FD) and curriculum integration of AI education (135). FD refers to the planned activities such as workshops, webinars and certifications that help faculty to keep their knowledge and skills abreast of the current trends. Such skill development will promote the integration of AI technology into teaching for instruction, feedback generation, and learning analytics and for research, while keeping them informed about evolving trends and subsequent policy revisions (125, 126, 136). Translation of these skills to initiate curricular changes such as redefining competencies for an era dominated by AI, developing teaching resources for students, applying AI tools to clinical care, interpreting AI-generated outputs, understanding limitations, and integrating digital tools into clinical decision-making, while being mindful of its ethical use, is the need of the hour (96). These competencies should align with broader educational policies defining digital literacy and its adaptability in healthcare training (30, 31, 122). AI-powered platforms including ChatGPT-assisted learning, intelligent tutoring systems like COMET, METEOR, CC-Cruiser, and decision-support systems such as WFO, consistently demonstrate improvement in students' knowledge acquisition, diagnostic accuracy, and clinical reasoning skills. A study testing the performance of ChatGPT in USMLE exams found it to be very effective with high lecle of concordance of its explanations of its choice of answers to the MCQs, suggesting that it could be of effective assistance in medical education, especially clinical decision making (9).

The third domain focuses on academic integrity and assessment reforms. There is a paucity of clearly defined policies in many universities regarding the acceptable use of generative AI in teaching, authorship, and assessment. This poses a significant challenge to academic integrity and the validity of assessments (128). Generative AI can produce detailed responses to the prompts, with analytical and critical assessments, making traditional written assignments an unreliable measure of student competency (136). Redesigning assessment methods to prioritize learning, clinical reasoning, and reflection related to activities could help in increasing the reliability of the assessment (136, 137). Therefore, the formulation of a clear system of checks and penalties guides students and promotes ethical AI use. Automated grading systems show high reliability, with agreement rates exceeding 85%–90% compared to human evaluators, while significantly reducing grading time (121). However, the feedback provided by the AI must be personalized and humanized by the educator. A meta-analysis found that AI-enhanced PBL or CBL improved knowledge acquisition (121). ChatGPT-assisted PBL significantly improved theoretical examination performance, interviewing skills, clinical judgment, and overall clinical competence (109). Personalized MeSH-guided feedback generated by ChatGPT-4o improved radiographic diagnostic performance more than traditional faculty feedback (110). It is important that faculty oversight is mandated to ensure the validity and accuracy of AI generated feedback (109). These findings reflect a movement toward adaptive assessment systems that identify learner gaps, produce tailored remediation materials, and continuously track performance. While technology enhanced assessment is promising, it also introduces challenges. It risks overreliance on automated tools and, therefore, a potential loss of critical thinking and humanistic competencies (109). Technology advanced assessments require hardware and training time. Resource constraints when implementing advanced systems like VR, haptic sim, or complex AI engines can pose significant challenges (121). Critical skills, such as creating high-quality MCQs and skills to integrate generative AI tasks in education, may require frequent or a series of workshops to be most beneficial, although single workshop models for innovative topics, such as the application of AI for education, serve to incite interest (138–140). Therefore, assessment transformation requires thoughtful implementation paired with faculty development and institutional support.

The final domain focuses on leadership and strategic change management. Transformational leaders are the need of the hour for adoption of AI technologies in healthcare. A transformational leader is defined by the ability to inspire innovation, adaptability and motivate learning and creativity within teams (141). Change management by leaders involves a complex multidimensional approach requiring leadership to align strategic plans to organizational goals, policy considerations, promote collaboration, allocate technological and operational resources, and plan for risk management (141, 142). Leaders must amalgamate technical expertise, adaptive strategies, and effective interpersonal and communication skills to steer the course of the institute through a shifting landscape sculpted by complex and interdependent factors governing AI implementation (143). Collaboration between universities, industry, professional bodies, and national policymakers is essential for AI integration to align with workforce needs and regulatory standards (144). Leadership and administration play a significant role in fostering a culture of innovation while maintaining educational quality and public trust and meeting future demands of medical practice.

Institutions that demonstrate readiness and employ AI and digital technologies enhance efficiency by better resource allocation and management (145, 146). Using advanced digital technologies facilitates communication and improves local and global collaboration efforts of the institutes. Advanced digital educational technologies, such as virtual labs and AI-powered learning platforms, contribute to efficiency in teaching while providing an innovative learning experience for the students (147, 148). On the other hand, challenges may also be faced in incorporating digital technologies, such as over-reliance on AI, disparities in access to the technology, irresponsible utilization of patient data, and lack of academic integrity and plagiarism (145, 148–150).

### 4.1 Resistance to change: faculty perspectives and attributes

The readiness of faculty constitutes a pivotal element in the successful integration of AI in dental curricula. Resistance to change is multifaceted and shaped by cognitive, institutional, and cultural factors and is commonly rooted in inadequate familiarity with AI, apprehensions about its technical demands, and skepticism regarding its role in clinical practice (151). A considerable proportion of faculty in dentistry are rooted in traditional pedagogical frameworks, with limited exposure to AI applications in clinical contexts. Studies indicate that 61.9% of educators are not yet routinely using digital and AI tools in assessments, primarily due to gaps in training and familiarity (152). This can undermine their self-assurance in teaching AI-centric content. Compounding factors that can amplify this reticence include anxieties over technological dependability, ethical ramifications, and the prospective reconfiguration of professional responsibilities (32). Practical barriers might complicate the adoption of AI in dental education. With demanding schedules, faculty members are juggling extensive teaching, research, and clinical commitments, leaving limited time for interacting with emerging technologies or investing in professional upskills. Structured faculty development efforts are indispensable to foster sustainable AI integration (54).

Institutional culture profoundly affects receptivity and engagement to innovation. Organizations that deliver specialized training, champion experimental practices allowing room for errors during the transition period, while rewarding digital competencies, create fertile ground for adoption; rigid frameworks with insufficient support systems, on the other hand, tend to obstruct transformation (33). Overcoming resistance calls for a phased approach encompassing mentorship, sustained professional development, and gradual integration strategies harmonized with established instructional approaches.

## 5 Global perspectives, and equity and ethical considerations

The integration of AI into health professions education unlocks significant prospects, while it simultaneously underscores critical equity concerns. A substantial digital chasm separates high-income countries (HICs) and low- and middle-income countries (LMICs), particularly in terms of technological access, infrastructure, and educational resources (34). Within HIC settings, educational institutions possess the capacity to integrate AI into their curricula, facilitated by advanced infrastructure and financial support systems. LMICs often grapple with formidable barriers, including inadequate access to digital tools and competing healthcare priorities (153). Such institutional gaps directly impact the adoption and sustainability of innovative teaching approaches (33).

Equity-centric interventions are imperative to counteract these challenges. Scalable digital solutions, open-access educational resources, and international collaboration can effectively close gaps in accessibility and institutional capacity. Approaches such as tele-education and mobile learning are particularly relevant in resource-constrained settings, where traditional infrastructure may be limited (154). At the same time, ethical considerations must remain at the forefront. AI systems derived from non-representative datasets may inadvertently perpetuate or exacerbate existing health inequities when applied across diverse populations (155). Such risks of bias highlight the importance of contextual adaptation and inclusive data curation practices. A coordinated global approach is imperative to ensure that AI integration in health professions education is both effective and equitable. Such efforts should align curriculum reforms with broader public health priorities while supporting the development of institutional readiness and resources across diverse global settings.

In dental practice, as with other health professions, ethical practice is defined by a set of “values, rights, principles and norms” are established, that not only guide but also serve to critically evaluate the conduct of the practitioner (156). With rapid evolution of technology and AI based solutions in dentistry ethical considerations for decision making, patient autonomy and rights, and research for greater good while maintaining patient safety and privacy is essential (157). The responsible utilization and ethical integration of advanced digital technologies in dental education and clinical practice have been critically examined in recent years. A comprehensive scoping review analyzing 178 studies reported that although applications are rapidly expanding, only 12.4% of studies explicitly addressed ethical concerns, indicating a significant gap in ethical discourse despite widespread adoption (158).

Resistance among the health professions academic community for data sharing due to concerns regarding data security and privacy when collaborating between institutes or at a national level; though such large data sets generated improve the reliability of the solutions and the validity of results (159). Changes in policies need to be in effect not just at the institutional level but also at the national level to ensure equity in education and access to resources (20, 25). In dentistry too, data privacy and secure data sharing remain key concerns in the application of AI. Legal and technical procedures to maintain the privacy of extremely sensitive and heterogeneous dental datasets by anonymization and encryption of radiographs, CBCT scans, and electronic health records must be complied with to enable multi-institutional collaboration (160). Rokhshad et al. proposed a checklist and a framework for the use of AI in dentistry, identifying 11 principles, *i.e.,* privacy protection, diversity, transparency, wellness, solidarity, equity, prudence, law and governance, sustainable development, accountability and responsibility, respect of autonomy and decision-making. Analysis of ethical considerations emphasizes that the lack of interpretability in automated systems can reduce clinician confidence and create ambiguity when errors occur (119).

Among the major limitations affecting fairness and equity in dental AI is Algorithmic Bias. Fédération dentaire internationale (FDI) World Dental Federation, in its policy statement on AI in dentistry emphasizes that AI should reduce inequity and aim to improve the quality of the training data (161). Evidence from contemporary literature indicates that a lack of diversity across populations in AI training datasets can amplify existing disparities, leading to inconsistent diagnostic or educational outcomes (162). This is particularly critical in dentistry, where variations in anatomical and demographic factors influence clinical decision-making. Inclusion of diverse datasets among populations of various ethnic backgrounds and continuous validation and testing is necessary to ensure equitable performance across different patient groups.

Overall, literature suggests that integration of AI in dentistry is a multidimensional approach and needs strict human oversight to secure the data, avoid bias, mitigation, and ensure transparency. Technological advancements enhance dental education, accuracy of oral diagnosis, treatment planning, quality of care to the patients, and ensure ethical standards. Relevant definitions in this article have been included in Table 1.

**Table 1:** Definitions.

Domain	Definition	Citation
1. Infrastructure	Educational infrastructure comprises the facilities, technologies, services, policies, and support systems that enable the delivery, management, and continuous improvement of medical education programs (163).	PMID: 16799285
		DOI: 10.1097/01.ACM.0000232413.43142.8b
1.1 Spaces	Physical, virtual, and social environments in which learning occurs and that are intentionally designed to support educational activities, learner engagement, collaboration, and knowledge construction (164).	PMID: 25655659
		DOI: 10.3109/0142159X.2014.1001349
1.2 Simulation	“Simulation is the artificial representation of a complex real-world process with sufficient fidelity with the aim to facilitate learning through immersion, reflection, feedback, and practice minus the risks inherent in a similar real-life experience” (165).	PMID: 24623932
		doi: 10.1016/S0377-1237 (12)60040-9
1.3 Digital Platforms	Digital platforms are also called learning management systems. These are integrated technology-based systems that support the planning, delivery, management, assessment, and evaluation of educational activities by enabling access to learning resources, communication, collaboration, feedback, and learner tracking in online or blended learning environments (166).	PMID: 41402987
		doi: 10.1080/10872981.2025.2603805
1.4 Analytics	Learning analytics in medical education is the methodical measurement, collection, analysis, and interpretation of learner-generated and educational data to understand learning processes, monitor performance, provide feedback, personalize instruction, and optimize educational outcomes within medical training programs (167).	PMID: 39906079
2. Safe Learning Environment	A safe learning environment in medical education is an educational setting characterized by psychological safety, where learners feel respected, supported, and able to engage in learning activities, ask questions, admit errors, seek feedback, and express ideas without fear of embarrassment, humiliation, discrimination, or punitive consequences (81).	PMID: 23269291
		DOI: 10.1097/ACM.0b013e31827bfa14
2.1 Psychological Safety	Psychological safety (PS) is the belief that the environment is safe for risk taking (84).	PMID: 37266963
		DOI: 10.1080/0142159X.2023.2216863
2.2 Inclusion & Respect	“Inclusion is the institutional culture that promotes the diversity and uniqueness of each individual through practice, policy, and the development of cultural norms, and creates a high sense of belonging for all members within the organization”.	PMID: 39429511
		doi: 10.12688/mep.20515.2
	Respect is the expression of “inclusion of individuals with a wide range of backgrounds, identities, and life experience” (168, 169).	PMID: 32345871
		DOI: 10.1097/ACM.0000000000003215
2.3 Professional Identity Formation	“Professional identity formation is the transformative process through which one comes to think, act, and feel like a physician” (170).	PMID: 25054423
		DOI: 10.1097/ACM.0000000000000427
3. Faculty Development	“Faculty development has been defined as that broad range of activities that institutions use to renew or assist faculty in their roles (Centra, 1978), and includes initiatives designed to improve the performance of faculty members in teaching, research and administration” (171).	PMID: 17074699
		doi:10.1080/01421590600902976
3.1 Pedagogy	Pedagogy is educator centric method of teaching where teacher decides the curriculum, methods of learning, and assessment (172).	PMID: 33426105
3.2 Assessment	Assessment involves testing, measuring, collecting, and combining information, and providing feedback and criteria provide the basis and the framework for judgments or decisions (173).	PMID: 21345060
		DOI: 10.3109/0142159X.2011.551559
3.3 Feedback	Specific information about the comparison between a trainees observed performance and a standard, given with the intent to improve the trainees performance (174).	PMID: 18230092
		doi:10.1111/j.1365-2923.2007.02973.x
3.4 Role Modeling	“Role modelling is a powerful teaching tool for passing on the knowledge, skills, and values of the medical profession” (175).	PMID: 18369229
		doi:10.1136/bmj.39503.757847.BE
4. Governance & Policy	“Governance is a process by which curricular decisions are made and implemented, involving the structure of decision-making groups, formal and informal relationships among these groups and individuals responsible for implementation.”	PMID: 28640029
	Policies are defined as “formal, authoritative statements that establish rules, responsibilities, and procedures” (176).	DOI: 10.1097/ACM.0000000000001774
		10.1186/s41239-026-00602-z
4.1 AI ethics & Guidelines	AI ethics is a multidisciplinary field that studies how to optimize the beneficial impact of artificial intelligence (AI) while reducing risks and adverse outcomes (177).	https://www.ibm.com/think/topics/ai-ethics
4.2 Data Governance	Data governance means the processes, people, policies, practices, and technologies that govern the data lifecycle, with the goal of increasing trust, value, and equity while minimizing risks and harms. The Data Governance Toolkit was designed with this definition in mind (178).	https://www.unesco.org/en/en/data-governance-digital-age/about
4.3 Regulatory Compliance	The regulatory framework is designed to uphold high standards in educational quality and performance. One of the core mechanisms for assuring compliance is the inspection process conducted by relevant regulatory bodies (179).	https://generisonline.com/understanding-education-regulations-in-the-united-arab-emirates-a-comprehensive-overview
5. Curriculum Integration & Identify Training	The term curriculum integration (CI) refers to combining two or more subjects when teaching a topic. CI involves integrating the subject concepts, subject content (the facts or substantive knowledge), and subject competencies (or skills) developed in a topic (180).	https://theeducationhub.org.nz/an-introduction-to-curriculum-integration/
5.1 AI Literacy Training	AI literacy refers to a foundational conceptual understanding of AI (181).	10.1080/10494820.2025.2514372
5.2 Educational Resources	Education resources are materials and tools designed to support teaching and learning processes. These resources include textbooks, digital content, instructional guides, and multimedia tools that enhance educational experiences (182).	https://www.unesco.org/en/query-list/e/educational-resources
6. Academic Integrity	commitment to the fundamental principles and values—honesty, trust, fairness, respect, responsibility in learning, teaching, and research, and courage (183).	PMID: 35815317
6.1 Redesign Assessment	The assessing of academic or educational achievement. It includes all aspects of testing and test construction (184).	https://id.nlm.nih.gov/mesh/D004521
6.2 Authentic Evaluation	Works consisting of studies determining the effectiveness or utility of processes, personnel, and equipment (185)	http://id.nlm.nih.gov/mesh/D023362
6.3 Educational Resources	Identification, development, organization, or utilization of educational resources and the management of these processes. It is occasionally used also in a more limited sense to describe the use of equipment-oriented techniques or audiovisual aids in educational settings (185).	http://id.nlm.nih.gov/mesh/D018961
7. Leadership & Change	Leadership: The function of directing or controlling the actions or attitudes of an individual or group with more or less willing acquiescence of the followers (185).	http://id.nlm.nih.gov/mesh/D007857
7.1 Strategic Planning	A rigorous process which entails defining plans, decisions, and sequence of steps to be taken in the future (186).	http://id.nlm.nih.gov/mesh/D000070318
7.2 Resource Allocation	to review the arrangements for distributing capital and revenue to authorities, with a view to establishing a pattern of distribution responsive objectively, equitably and efficiently to relative need (187)	PMID: 19622159
7.3 Collaboration & Advocacy	Collaboration: Cooperative actions and ventures among health and health-related groups and organizations intended to improve health outcomes (188)	http://id.nlm.nih.gov/mesh/D000070516
	Advocacy:Health-care advocacy extends beyond the understanding of environmental, social, and economic factors that affect health. Patient advocacy is more than a role–it is a principle that governs the patient-physician relationship and the practice of medicine (189)	PMID: 9787754

## 6 Future directions and conclusion

Future directions should be aimed at advancing institutional readiness and faculty development to integrate AI in health professions education, keeping pace with its rapid progress. Institutions should invest in robust educational infrastructure, including digital platforms, simulation technologies, learning analytics, and supportive learning spaces. It is crucial to create psychologically safe and inclusive learning environments that promote professional identity formation, respect, collaboration, and learner engagement. It should also equip faculty with the pedagogical knowledge and technological competencies required to integrate AI effectively into curriculum design, teaching, assessment, and constructive feedback practices.

The organizational culture in health professions education should prioritize ethical governance, policy development, quality assurance, and secure data management to protect student and patient privacy. Policy makers and accrediting bodies must enforce regulatory compliance, and accountability, while adopting innovations. The development of implementation frameworks by policymakers and institutional leaders should provide policies and clear guidelines when using AI for research and to train AI models.

AI literacy in health professions, especially dental curricula, should be integrated from the ground up rather than as an afterthought. Faculty should take on the role of a facilitator, and AI will serve as a personal tutor. Assessment should be revisited to ensure that there is no compromise in critical thinking and clinical judgement among students, particularly when they interact with AI tools. Both students and practicing clinicians, including dentists, should be encouraged to use AI as a decision support tool while avoiding over-reliance on AI in clinical practice. To promote equity and trustworthiness of AI applications in dentistry, dental AI systems must be trained on diverse datasets to reduce bias, ensuring they represent varied patient populations and clinical scenarios. A clinician-led interdisciplinary collaboration with industry partners, engineers, and data scientists, is of utmost importance to ensure clinical relevance and educational value, as most AI tools in the dentistry are developed by engineers without any medical background.

Therefore, a successful integration of AI technology and its sustainability in health professions education depends on institutional readiness, faculty development, curriculum innovation, educational infrastructure, safe learning environment, assessment of reforms, ethical governance, leadership, and academic integrity. Transformative strategies grounded in strategic planning, resource allocation, collaboration, continuous faculty empowerment, and global equity can ensure the preparation of competent, adaptable, and socially responsible health professionals. By maintaining human oversight and professional judgement at the center of higher education, institutions can leverage AI as a catalyst for modernization while preserving the core values of equity, professionalism, and patient care.
