# Correction: Faculty development and institutional readiness for incorporating AI in health professions education—a narrative review

**DOI:** 10.3389/fdmed.2026.1986847

**Published:** 2026-09-11

**Authors:** Prathibha Prasad, Mahinour Amin, Elizabeth Fitriana Sari, Lovely M. Annamma, Jayaraj Narayanan, Dinesh Yasothkumar, Mehzabin Ahmed

**Affiliations:** 1 Department of Basic Medical and Dental Sciences, College of Dentistry, Ajman University, Ajman, United Arab Emirates; 2 Director of Teaching and Learning Center, Ajman University, Ajman, United Arab Emirates; 3 Adjunct Faculty, Dental Research Cell, Dr D. Y. Patil Dental College & Hospital, Dr D. Y. Patil Vidyapeeth (Deemed to be University), Pune, Maharashtra, India; 4 Consultant Oral and Maxillofacial Pathologist, Dentscope Advanced Dental Studio, Chennai, India; 5 Biomedical Sciences Department, College of Medicine, Gulf Medical University, Ajman, United Arab Emirates

**Keywords:** artificial intelligence, dental education, ethics, faculty development, global perspectives, health professions education, higher education institutions

Affiliation “Faculty, College of Dentistry, Ajman University, Ajman, United Arab Emirates” was erroneously included in the paper. This affiliation has now been removed for author Lovely M. Annamma.

The original version of this article has been updated.

